# Supplementary material for: Deep spectral improvement for unsupervised image instance segmentation
Source: PLoS One. 2024 Oct 7;19(10):e0307432. doi: 10.1371/journal.pone.0307432 (PMC11458003; doi:10.1371/journal.pone.0307432)
Supplement: S1 Table — (PDF) [file pone.0307432.s001.pdf]

Youtube-VIS 2019		
**Value of M**	**w post-processing**	**w/o post-processing**
M = C	53.9	50.68
M = 3C/4	53.89	50.74
M = C/2	55.79	52.85
M = C/3	**56.84**	**54.13**
M = C/5	56.69	53.91
M = C/7	55.93	52.91
M = C/9	55.23	52
M = C/11	54.33	50.92
**PascalVoc 2012**		
**Value of M**	**w post-processing**	**w/o post-processing**
M = C	58.66	57.68
M = 3C/4	58.59	57.62
M = C/2	58.32	57.37
M = C/3	58.56	57.76
M = C/5	**59.69**	**58.81**
M = C/7	59.01	58.22
M = C/9	58.77	57.70
M = C/11	58.30	57.13
**Davis 2016**		
**Value of M**	**w post-processing**	**w/o post-processing**
M = C	50.68	46.63
M = 3C/4	51.51	47.41
M = C/2	54.77	50.88
M = C/3	**55.89**	**52.00**
M = C/5	55.32	51.07
M = C/7	54.21	46.61
M = C/9	53.23	48.15
M = C/11	51.90	46.56
