# Supplementary material for: Deep spectral improvement for unsupervised image instance segmentation
Source: PLoS One. 2024 Oct 7;19(10):e0307432. doi: 10.1371/journal.pone.0307432 (PMC11458003; doi:10.1371/journal.pone.0307432)
Supplement: S2 Table — (PDF) [file pone.0307432.s002.pdf]

**Metric**	**Youtube-VIS 2019 mIoU (%)**	**OVIS mIoU (%)**
Mahalanobis	25.27	25.91
L1	31.53	33.74
Dot product	32.71	33.34
L2	32.77	34.92
Chebyshev	33.09	34.63
Cosine	33.56	35.57
Correlation	34.08	35.93
Braycurtis	34.14	35.99
**BoC**	**34.41**	**36.14**
