# Supplementary material for: Deep spectral improvement for unsupervised image instance segmentation
Source: PLoS One. 2024 Oct 7;19(10):e0307432. doi: 10.1371/journal.pone.0307432 (PMC11458003; doi:10.1371/journal.pone.0307432)
Supplement: S3 Table — (PDF) [file pone.0307432.s003.pdf]

**Metric**	**MBOR 0.01-0.14**	**MBOR 0.14-0.34**	**MBOR $\geq$ 0.34**
Mahalanobis	24.46	27.20	26.86
L1	31.92	34.18	30.25
Dot product	33.50	35.25	30.69
L2	33.42	35.26	30.99
Chebyshev	33.65	35.91	31.31
Cosine	34.03	36.26	31.38
Correlation	34.76	36.67	31.64
Braycurtis	34.67	37.06	31.88
**BoC**	**34.94**	**37.38**	**32.33**
