# Supplementary material for: Deep spectral improvement for unsupervised image instance segmentation
Source: PLoS One. 2024 Oct 7;19(10):e0307432. doi: 10.1371/journal.pone.0307432 (PMC11458003; doi:10.1371/journal.pone.0307432)
Supplement: S4 Table — (PDF) [file pone.0307432.s004.pdf]

**Metric**	**Ratio 1.26-1.64**	**Ratio ≥1.64**
Mahalanobis	26.57	23.98
L1	32.10	30.97
Dot product	34.23	31.20
L2	33.54	31.99
Chebyshev	34.08	32.10
Cosine	34.23	32.90
Correlation	34.98	33.18
Braycurtis	34.91	33.38
**BoC**	**35.10**	**33.71**
