# Supplementary material for: Deep spectral improvement for unsupervised image instance segmentation
Source: PLoS One. 2024 Oct 7;19(10):e0307432. doi: 10.1371/journal.pone.0307432 (PMC11458003; doi:10.1371/journal.pone.0307432)
Supplement: S5 Table — (PDF) [file pone.0307432.s005.pdf]

**Metric**	**Distance <0.29**	**Distance 0.29-0.41**	**Distance ≥0.41**
Mahalanobis	26.29	25.86	23.67
L1	31.63	32.59	30.38
Dot product	33.12	33.51	31.50
L2	32.68	33.63	31.99
Chebyshev	33.34	34.04	31.87
Cosine	33.27	34.66	32.76
Correlation	33.82	35.09	33.33
Braycurtis	33.85	35.27	33.31
**BoC**	**34.25**	**35.58**	**33.39**
