# Supplementary material for: Deep spectral improvement for unsupervised image instance segmentation
Source: PLoS One. 2024 Oct 7;19(10):e0307432. doi: 10.1371/journal.pone.0307432 (PMC11458003; doi:10.1371/journal.pone.0307432)
Supplement: S6 Table — (PDF) [file pone.0307432.s006.pdf]

**Metric**	**smoothness** $< \mathbf{0.13}$	**smoothness 0.13-0.68**	**smoothness** $\geq \mathbf{0.68}$
Mahalanobis	23.50	24.50	27.82
L1	30.83	31.54	32.23
Dot product	32.46	32.35	33.32
L2	32.32	32.76	33.23
Chebyshev	32.85	32.99	33.42
Cosine	32.95	33.95	33.79
Correlation	33.74	34.29	34.20
Braycurtis	33.52	34.31	34.59
**BoC**	**33.99**	**34.47**	**34.76**
