# Supplementary material for: Deep spectral improvement for unsupervised image instance segmentation
Source: PLoS One. 2024 Oct 7;19(10):e0307432. doi: 10.1371/journal.pone.0307432 (PMC11458003; doi:10.1371/journal.pone.0307432)
Supplement: S7 Table — (PDF) [file pone.0307432.s007.pdf]

NCR	DCR	BoC	mIoU (%)
			31.75
✓			32.92
✓	✓		32.70
		✓	30.50
✓		✓	33.62
✓	✓	✓	**34.41**
